# Ovariotomy in a Child Twenty Months Old

**Published:** 1885-02

**Authors:** 


*Ovariotomy in a Child Twenty Months Old.*—After a previous puncture, Romer performed laparotomy under a carbolized spray and extirpated an ovarian cyst, the size of a child's head. The cure occurred without any disturbance. This operation is unique in the literature, as so far such a splendid ovariotomy in a child of such atender age, was never practiced, with equal success.—*Berl. Klin. Wochenschrift.*

